# Trigonelline recovers memory function in Alzheimer’s disease model mice: evidence of brain penetration and target molecule

**DOI:** 10.1038/s41598-020-73514-1

**Published:** 2020-10-02

**Authors:** Mai M. Farid, Ximeng Yang, Tomoharu Kuboyama, Chihiro Tohda

**Affiliations:** 1 Section of Neuromedical Science, Division of Bioscience, Institute of Natural Medicine, University of Toyama, 2630 Sugitani, Toyama, 930-0194 Japan; 2 Pharmaceutical and Drug Industry Division, Department of Phytochemistry and Plant Systematics, National Research Centre, Giza, Egypt

**Keywords:** Pharmacology, Drug development

## Abstract

Trigonelline (TGN; 1-methylpyridin-1-ium-3-carboxylate) is a widely distributed alkaloid derived from plants. Since we previously found a neurite outgrowth effect of TGN, we hypothesised that TGN might help to improve memory deficits. Here, the efficacy of TGN in restoring amyloid β (Aβ)-induced axonal degeneration and in improving memory function was investigated in Alzheimer’s disease 5XFAD model mice that overexpress mutated APP and PS1 genes. Exposure of Aβ25-35 for 3 days induced atrophy of axons and dendrites. Post treatment of TGN recovered the lengths of axons and dendrites. Following oral administration of TGN in mice, TGN itself was detected in the plasma and cerebral cortex. Oral administration of TGN to 5XFAD mice for 14 days showed significant improvement in object recognition memory (*P* < 0.001) and object location memory (*P* < 0.01). TGN administration also normalised neurofilament light levels in the cerebral cortex (*P* < 0.05), which is an axonal damage-associated biomarker. Analysis of target proteins of TGN in neurons by a drug affinity responsive target stability (DARTS) method identified that creatine kinase B-type (CKB) is a direct binding protein of TGN. Treatment with a CKB inhibitor cancelled the TGN-induced axonal and dendritic growth. In conclusion, we found for the first time that TGN penetrates the brain and may activate CKB, leading to axonal formation. This study shows the potential of TGN as a new drug candidate, and a new target molecule, CKB, in memory recovery signalling.

## Introduction

Alzheimer’s disease (AD) is a progressive neurodegenerative disorder that mainly affects older people. AD is characterized by deposition of amyloid β (Aβ) in the brain, therefore decades of research have focused on several agents that reduce the Aβ level. Unfortunately. these agents have failed in phase 2 or 3 clinical trials^1^, suggesting that regaining memory function in spite of a decline in Aβ in the brain is difficult. We hypothesized that reconstructing the damaged neuronal network may help recover neuronal functions.

Trigonelline (TGN) is a major pyridine alkaloid component and a methylation product of niacin (vitamin B3), first isolated from *Trigonella foenum*-*graecum*^2^*.* It also exists in many other plant species such as *Glycine max*, *Allium sepapea,* and *Coffea* spp^3^. Furthermore, many common foods contain TGN such as barley, cantaloupes, corn, onions, soybeans, peas, and tomatoes^4^. Numerous reports on the biological activity of TGN have mentioned its role in protecting the liver and heart and in the treatment of hypercholesterolaemia, hyperglycaemia, and central nervous system disorders^5^. Only one report has suggested the potential role of TGN as an anti-Alzheimer’s drug. Microinjection of amyloid β (1-40) (Aβ1-40) to the hippocampal CA1 induced memory loss in rats. In addition, peroral pre-administration of 100 mg/kg TGN attenuated neuronal death and oxidative stress-related changes in CA1, as well as memory dysfunction^6^. However, anti-AD effect of TGN has not investigated yet in a well-established AD model like a transgenic mouse.

The antioxidative effects of TGN in the brain have also been demonstrated in other cognitive disorder models, but not necessary AD model^7^. In lipopolysaccharide-induced memory loss model, peroral pre-administration of TGN at 50 and 100 mg/kg normalised levels of superoxide dismutase, reduced glutathione, malonaldehyde, tumour necrosis factor-alpha, interleukin 6, and brain-derived neurotrophic factor (BDNF) in the brain, and memory function. An in vitro study also focussed on the neuroprotective effect of TGN and suggested an antioxidative effect as the primary effect of TGN^8^.

In contrast, we have investigated the drug activities of recovering and/or holding back neuritic atrophy and loss of synapses because these are located upstream of neuronic death in the Aβ cascade^9,10^. Compounds that showed a restoring effect on Aβ-induced axonal atrophy also recovered memory impairment in an AD model mouse, 5XFAD^11–13^. The neurite outgrowth effect of TGN was first found in human neuroblastoma SK-N-SH cells^14^ and Aβ25-35-treated primary cultured cortical neurons^15^. Therefore, we supposed TGN had a potency of neural network repairing and aimed to clarify whether TGN improved memory dysfunction and axonal damage in 5XFAD mice, which overexpress mutated APP and PS1 genes. In addition, we investigated the brain penetration of TGN, which has not been previously reported, although absorption in the blood has been indicated in several pharmacokinetic studies^16–18^. Furthermore, the mechanism of action of TGN, other than antioxidative stress, was examined.

## Materials and methods

All experiments were performed in accordance with the Guidelines for the Care and Use of Laboratory Animals of the University of Toyama, Japan. The Committee for Animal Care and Use at the Sugitani Campus of the University of Toyama approved the study protocols. The approval number for the animal experiments was A2017INM-1 and G2018INM-2. All efforts were made to minimise the number of animals used.

### Primary culture

Primary culture was performed as previously we did^11,12^. Embryos were removed from ddY mice (Shizuoka, Japan) at 14 days of gestation. The cortices were dissected, and the dura mater was removed. The tissue was minced, dissociated, and grown in cultures with neurobasal medium (Invitrogen, Grand Island, New York, USA) that included 12% B-27 supplement (Invitrogen), 0.6% D-glucose, and 2 mM of L-glutamine on 8-well chamber slides (Falcon, Franklin Lakes, New Jersey, USA) coated with 5 µg/mL poly-D-lysine at 37 °C in a humidified incubator with 10% CO_2_. The seeding cell density was 2.2 × 10^4^ cells/cm^2^.

### Immunocytochemistry and measurement of axonal and dendritic densities

Immunohistochemistry was performed as previously we did^11,12^. After the cortical neurones were cultured for 3 days, Aβ25-35 (10 µM) was simultaneously treated with TGN (at doses of 1 and 10 µM) (Cat. No. 11904, Cayman Chemical, Ann Arbor, Michigan, USA) for 4 days. Then, axons and dendrites were immunostained, and the neurones were fixed with 4% paraformaldehyde (Cat. No. 162-16065, Wako Pure Chemical Industries) for 90 min and were immunostained with a monoclonal antibody. Phosphorylated neurofilament-H (pNF-H; dilution 1:250, Cat. No. 835601, SMI-35R, Covance, Dedham, Massachusetts, USA) was used as an axonal marker. A polyclonal antibody against microtubule-associated protein 2 (MAP2, dilution 1:2000, Cat. No. ab32454, Abcam, Cambridge, UK) was used as a dendritic marker. The first antibody reaction was performed in phosphate buffered-solution (PBS) containing 1% normal goat serum (Cat. No. 143-06561, Wako Pure Chemical Industries) and 0.3% Triton X-100 (Cat. No. 168-11805, Wako Pure Chemical Industries). Alexa Fluor 488-conjugated goat anti-mouse immunoglobulin G (IgG; dilution 1:300, Cat. No. A-11029, Thermo Fisher Scientific, Waltham, Massachusetts, USA) and Alexa Fluor 594-conjugated goat anti-rabbit IgG (dilution 1:300, Cat. No. A-11012, Thermo Fisher Scientific) was used as the secondary antibody. Nuclear counterstaining was performed using DAPI (1 μg/mL, Cat. No. D9542, Sigma-Aldrich, St. Louis, Missouri, USA). Fluorescent images were captured with a 10 × objective dry lens (Plan-Apochromat, Carl Zeiss, Oberkochen, Germany) using a charge-coupled device camera (AxioCamMRm, binning set at 1 × 1, Carl Zeiss) on an inverted microscope (Axio Observer Z1, Carl Zeiss). A total of 12–20 images were captured per treatment. The lengths of the pNF-H-positive axons and MAP2-positive dendrites were measured using a MetaMorph analyser (Molecular Devices, Sunnyvale, California, USA), which automatically traced and measured the neurite length without measuring the cell bodies. The sum of the axons or dendrite length was divided by the number of MAP2-positive neurones, which were counted using the MetaMorph analyser.

### Animal studies

Transgenic mice (5XFAD) were obtained from the Jackson Laboratory (Bar Harbor, Maine, USA) and maintained by crossing hemizygous transgenic mice with B6/SJL F1 breeders. To investigate the effect of TGN on 5XFAD mice, we used hemizygous transgenic 5XFAD mice (females, 9–11 months old) and non-transgenic wild-type littermate mice (female, 9–11 months old). All mice were housed with free access to food and water and kept in a controlled environment (22 ± 2 °C, 12-h light/dark cycle starting at 7:00 am).

### Behavioural test

TGN was suspended in distilled water and was orally administered and vehicle solution (distilled water) by gavage once per day for 14 days (10 mg/kg/day). After 14 days of compound administration, an open-field locomotion test was performed. Mice were individually habituated to an open-field box composed of polyvinyl chloride (30 cm × 40 cm: height, 36 cm) for 10 min. Their paths were tracked using a digital camera system. The distance moved over a 10 min period was considered to be the locomotor activity and was analysed with EthoVision version 3.0 (Noldus, Wageningen, Netherlands). At days 16 and 17, object recognition memory and object location memory tests were performed, respectively, as described previously^11,12^.

Mice were divided in each group; wild type/veh (n = 6), 5XFAD/veh (n = 7), and 5XFAD/TGN (10 mg/kg) (n = 7). All tests were carried out in a dimly illuminated room (90 lx).

### Western blotting

The mouse brain cortex or hippocampus was homogenised with M-PER (Thermo Fisher Scientific) containing 1 × Halt protease & phosphatase inhibitor cocktail (Thermo Fisher Scientific). The brain lysate (10 µg/lane) was loaded on 10% sodium dodecyl sulphate–polyacrylamide gel electrophoresis (SDS-PAGE) for the western blot analysis. After blocking the membrane with 5% skim milk (Wako Pure Chemical Industries, Japan) in 0.1% Tween tris-buffered saline for 30 min at room temperature, the 1st antibody, rabbit neurofilament-L (1:1000, Cat. No. 2837, Cell Signalling Technology, Danvers, Massachusetts, USA), and mouse Glyceraldehyde-3-phosphate dehydrogenase (GAPDH) (1:1000, Cat. No. 016-25523, Wako, Osaka, Japan), mouse synaptophysin (1:1000, clone SVP-38, Cat. No. S5768, Sigma-Aldrich), mouse purified anti-β-Amyloid, 1–16 antibody (1:1000, clone 6E10, Cat. No. 803001, BioLegend, San Diego, California, USA) and 2nd antibody, HRP-conjugated anti-rabbit IgG (1:2000), or HRP-conjugated anti-mouse IgG (1:2000) were used (Santa Cruz Biotechnology, Dallas, Texas, USA). Amersham ECL Western Blotting Detection Reagent (Sigma-Aldrich) was used for the detection of bands according to the manufacturer’ s protocol.

### Absorption and brain penetration of TGN

TGN or vehicle solution (distilled water) was orally administered to wild-type mice (9–11 months old, female, n = 3–4) at a concentration of 500 mg/kg. Liquid chromatography-mass spectrometry (LC–MS/MS) was used to determine whether TGN could be detected in the brain 5 min, 30 min, 6 h, and 24 h after the oral administration of 500 mg/kg TGN. The mice were then euthanised, and blood was collected. Plasma was obtained after centrifugation of blood at 11,000 g for 10 min at 4 °C. The brain cortex was dissected following perfusion with saline. Plasma (100 µL) was extracted with methanol, dried, and resolubilised in 100 µL methanol. The brain cortex was homogenised and extracted with methanol, dried, and resolubilised in 100 µL methanol before loading.

An Accela HPLC system (Thermo Fisher Scientific, Bremen, Germany) equipped with an aquaternary pump, a built-in solvent degasser, column compartment, and thermostated auto-sampler (PAL system) was adopted. The separation was achieved on an InertSustain AQ-C18 column (4.6 mm ID × 50 mm, 5 µm) under linear gradient elution with solvents A (0.01% formic acid in water) and B (methanol). The gradient profile started with isocratic elution of 2% B for 0 min, then linearly changed to 5% B for 5 min, then changed to 35% B for 10 min, followed by 15 min of isocratic elution to return to 2% B and 20 min elution using the same 2% B. The column temperature was set at 40 °C. The injection volume was 5 µL for each sample. A standard curve for TGN measurement was made. The Accela HPLC system was hyphenated with the LTQ/Orbitrap XL mass spectrometer. MS data acquisition was achieved using an LTQ/Orbitrap XL mass spectrometer (Thermo Fisher Scientific, Waltham, MA) with an electrospray ionisation (ESI) probe in positive ion mode. The auto-tuned condition was determined by direct infusion of the standard chemical, TGN, where the capillary voltage and temperature were set to − 50 V and 330 °C, the spray voltage was 3.0 kV, and the tube voltage was set to − 130 V. Sheath gas (N2) with a flow rate of 50 arbitrary units was used, and the auxiliary gas flow was set to 10 arbitrary units. The acquisition mass range was selected at m/z 100–2000 with a resolution of 30,000. Higher collision-induced dissociation (HCD)-based ESI-Orbitrap-MS/MS, collision-induced dissociation (CID)-based ESI-LTQ-MSn (n = 1–4), and ESI-Orbitrap-MS full-scan were carried out in the MS/MS similarity networking. Both HCD and CID were conducted in a data-dependent acquisition mode. The HCD energy was optimised and set to 40% normalised collision energy (NCE). The CID energy was set to 35% NCE in every dissociation stage. All spectra were acquired and processed using LTQ Xcalibur and Trace Finder software (Thermo Fisher Scientific, Waltham, Massachusetts, USA).

### A drug affinity responsive target stability (DARTS) analysis

The lysate was prepared from primary cultured mouse cortical neurones (ddY, E14), with a concentration of 2 × 10^6^ cells/ 10-cm dish. After being washed three times with 1 × PBS, neuronic cells were homogenised with M-PER (Thermo Scientific) containing a × 100 protease inhibitor cocktail (Thermo Scientific). The brain lysate (5 µg) was added to 2 µM and 100 µM TGN or vehicle solution and incubated for 30 min at room temperature. The mixture was proteolyzed using different concentrations of thermolysin (0.005, 0.05, 0.5, and 5 µg) (Sigma-Aldrich) in reaction buffer [50 mM Tris–HCl (pH 8.0), 50 mM NaCl, 10 mM CaCl_2_] for 30 min at 37 °C. To stop proteolysis, 0.5 M ethylenediaminetetraacetic acid (pH 8.0) was added to each sample in a 1:10 ratio, and then 12% SDS-PAGE was prepared. The proteins in the gels were silver stained for visualisation using a SilverQuest Kit (Invitrogen, Carlsbad, California, USA). The band that showed a decrease in intensity in the TGN-treated lysate compared to the vehicle-treated lysate was cut out and examined in a nano liquid chromatography-tandem mass spectrometry (LC–MS/MS) system (Japan Bio Services, Saitama, Japan). A candidate protein from the electrophoresis band was identified using the MASCOT and spectrum data.

### Characterisation of creatine kinase B-type binding to TGN in vitro

5-Isothiocyanato-2-benzofuranyl-2-imidazoline (BU99006, Santa Cruz Biotechnology) as an inhibitor of creatine kinase B-type (10 and 20 µM) was prepared by dissolving it in dimethyl sulfoxide (10 mg/mL, with heating to 60 °C). After the cortical neurones were cultured for 3 days, cells were treated with TGN (at doses of 1 and 10 µM) with or without BU99006 for 4 days. Then, the axons and dendrites were immunostained and analysed using the same method described previously.

### Statistical analysis

Data are expressed as the mean ± standard error. GraphPad Prism version 6 (GraphPad Software, San Diego, California, USA) was used to perform a one-way analysis of variance (ANOVA) with post hoc Dunnett’s test or Bonferroni’s multiple comparison test, and repeated measures two-way ANOVA with post hoc Bonferroni’s test. A *P* value of < 0.05 was considered statistically significant.

## Results

### TGN significantly ameliorated axonal and dendrite atrophy in Aβ-treated neurones

**Figure 1:**
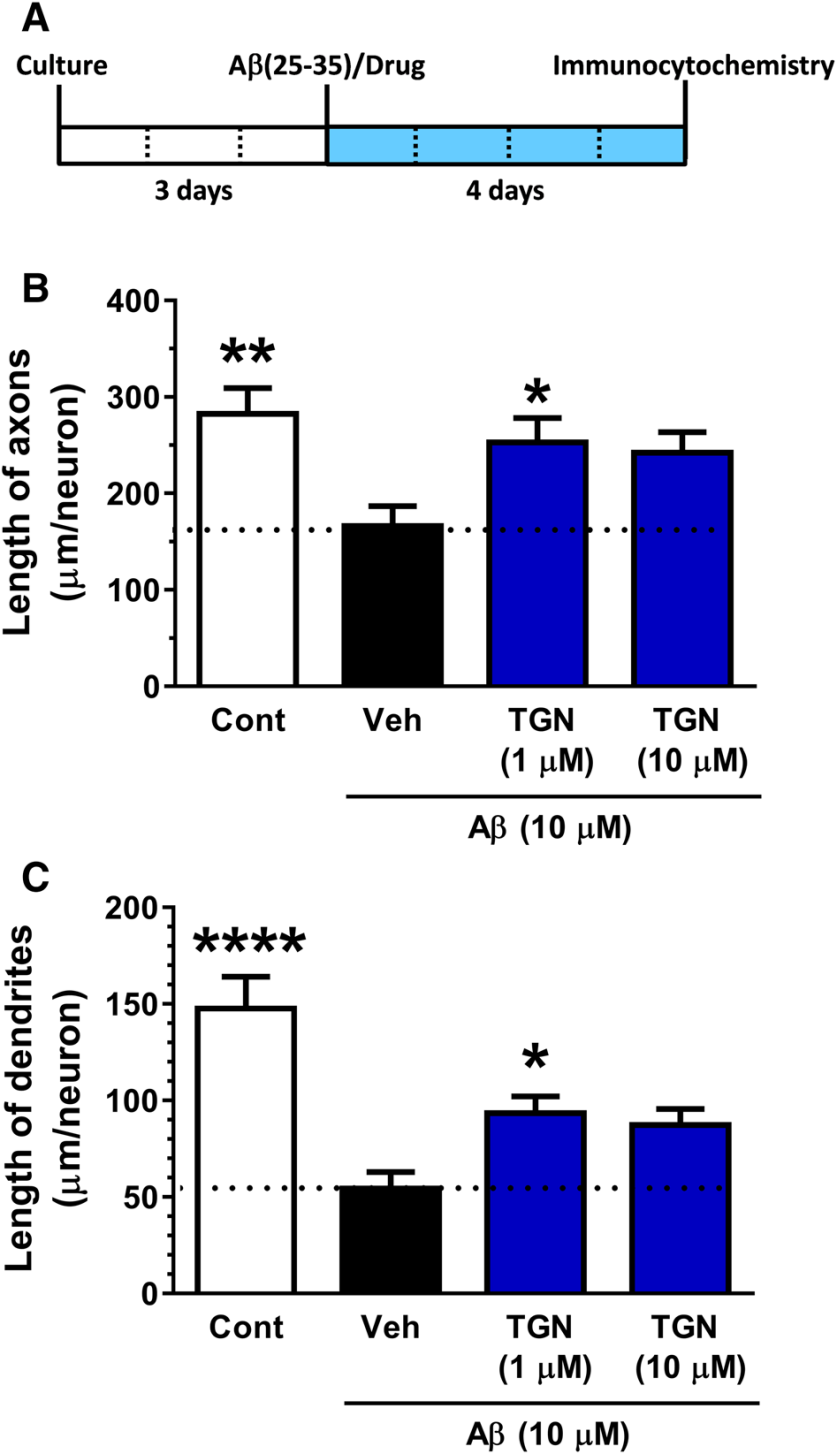
Effects of trigonelline (TGN) on axonal and dendrite growth in amyloid β (Aβ)-treated cortical neurones. (**A**) Time course of the experiment (**B**,**C**) Mouse cortical neurones were cultured for three days and then treated with Aβ25-35 (10 µM) with or without TGN (1 or 10 µM) or vehicle solution. After 4 days of treatment, the axons and dendrites were immunostained. Phosphorylated neurofilament-H (pNF-H) and microtubule-associated protein 2 (MAP2) were stained as axonal and dendritic markers, respectively. The statistical analysis was performed using a one-way analysis of variance (ANOVA) and post hoc Dunnett’s test, *P < 0.05, **P < 0.01, ****P < 0.0001 versus Aβ25-35 /Veh, n = 18–19 areas.

Three days after the cortical neurones were cultured, treatment of TGN at doses of 1 µM and 10 µM with Aβ25-35 (10 µM) was started. Four days after the treatment, axon and dendrite lengths were quantified (Fig. 1A). TGN (1 µM) significantly protected axonal and dendrite atrophies (Fig. 1B,C). Effects of TGN (1 µM) seemed to reach the plateau.

### Quantification of TGN in the blood and brain by LC–MS/MS indicates penetration to the brain after oral administration

**Figure 2:**
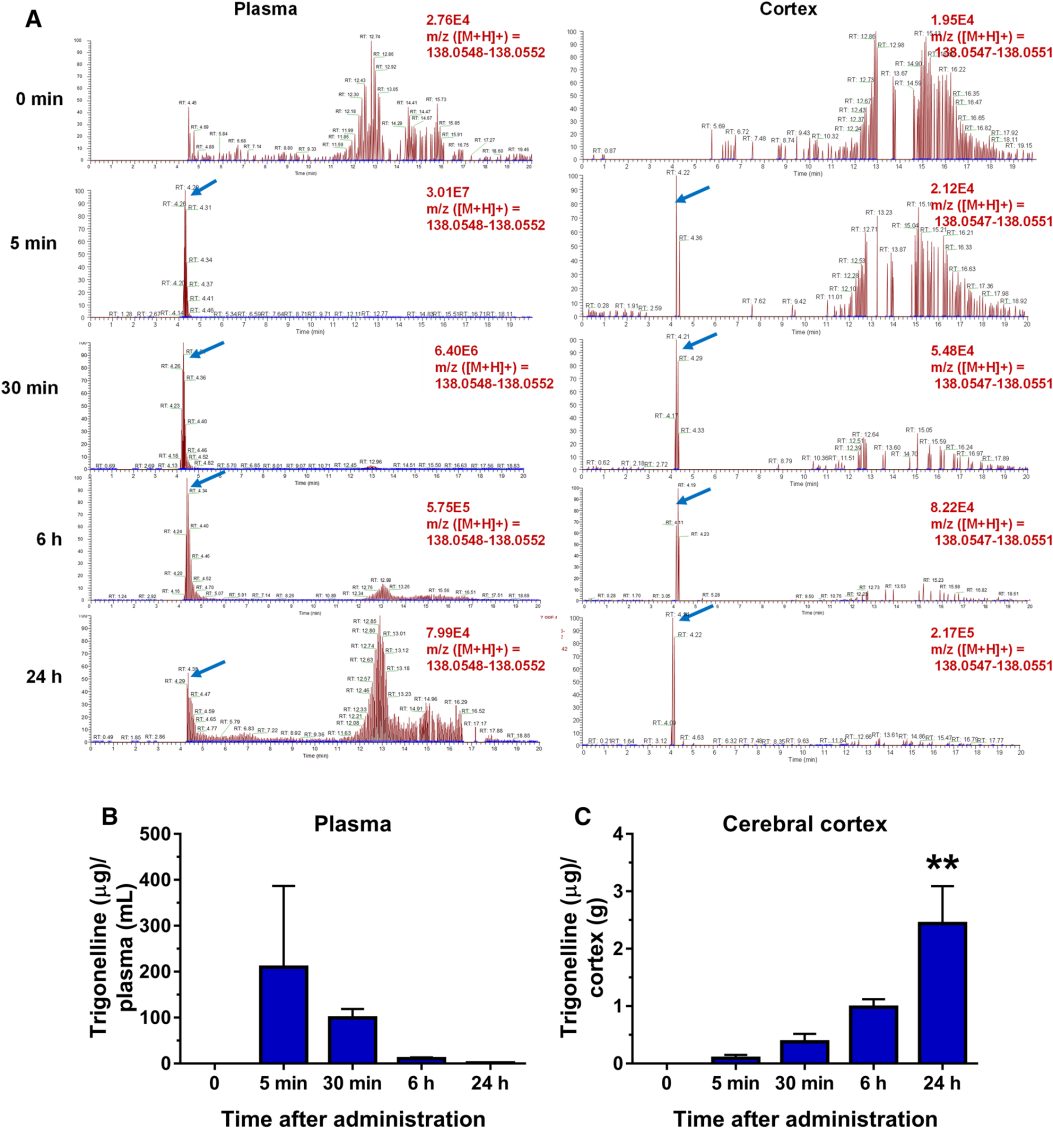
The absorbance of trigonelline (TGN) in the blood and cerebral cortex after the oral administration of TGN (500 mg/kg) or vehicle solution (water) was administered a single dose and sacrificed 5 min, 30 min, 6 h, and 24 h after treatment. The plasma and cerebral cortex were extracted with methanol and subjected to liquid chromatography-mass spectrometry (LC–MS/MS) analysis. (**A**) Detection of TGN in the plasma and cortex at 5 min, 30 min, 6 h, and 24 h after TGN treatment. The blue arrows indicate the TGN peaks. (**B**,**C**) TGN amounts were quantified in the plasma (**B**) and cerebral cortex (**C**). The statistical analysis was performed using a one-way analysis of variance (ANOVA) and post hoc Dunnett’s test, **P < 0.01 versus 0 min, n = 3 mice.

Next, we used LC–MS/MS to determine whether TGN could be detected in the blood and brain 5 min, 30 min, 6 h, and 24 h after the oral administration of 500 mg/kg TGN. Using the high-accuracy quasi-molecular ion ([M + H] +) and a mass error of ± 1 mmu, we detected TGN by comparing the MS–MS data and fragmentation patterns with a reference standard. The TGN molecular weight was 137.05 and could be detected by the positive mode at *m/z* 138.0549. The retention time and fragmentation pattern of TGN are shown in Fig. 2A. We confirmed that TGN was detected in the plasma and cortex from 5 min. After this, the TGN concentration in the plasma peaked 5 min after administration and then gradually decreased at 30 min, 6 h, and 24 h (Fig. 2B). In the cerebral cortex, the amount of TGN started to increase from 5 min and continuously accumulated to 24 h (Fig. 2C). The reason for the administration of a high dose of TGN was to overcome the detectable limitations of compounds by LC–MS/MS^12^.

### Memory improvement by oral administration of TGN

**Figure 3:**
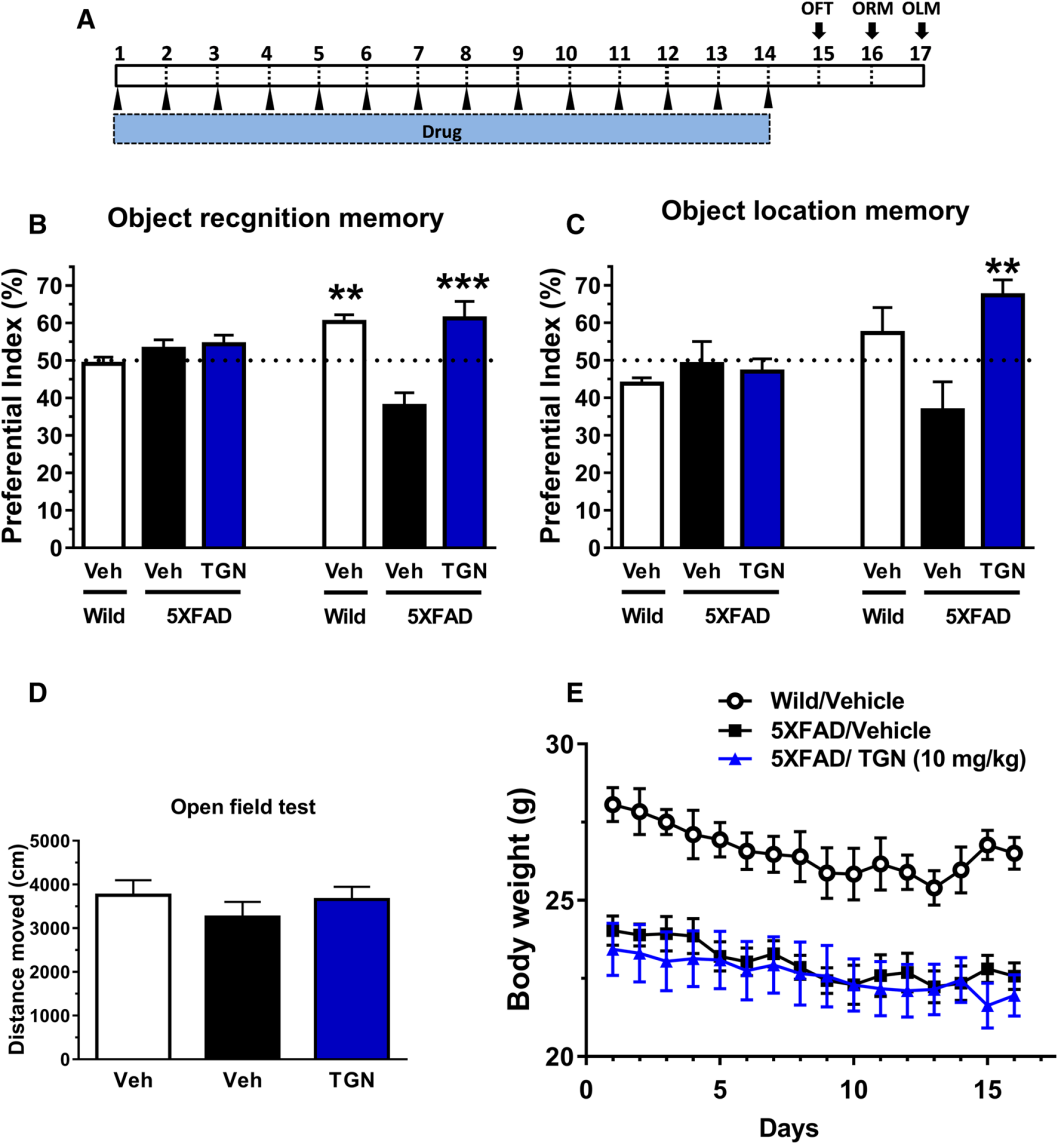
Effects of the oral administration of trigonelline (TGN) on object recognition memory and object location memory deficits in 5XFAD mice (**A**) Time course of the experiments. TGN (10 mg/kg/day, p.o.) or vehicle solution (physiological saline) was administered for 14 days to mice (female, 9–11 months old). The day after the last administration, an open field test was carried out. Two days and three days later, object recognition and object location tests were carried out, respectively. (**B**,**C**) The preferential indices of the training and test sessions in the object recognition (**B**) and object location tests (**C**) are shown. *P < 0.05, **P < 0.01 versus vehicle-treated 5XFAD mice. A one-way analysis of variance (ANOVA) with post hoc Dunnett’s test, n = 6—7 mice). (**D**) Distance moved over 10 min in the open field test to evaluate locomotion. (**E**) Bodyweight of the mice during the entire experimental period is shown. A repeated measures two-way ANOVA revealed no significant differences among the four groups.

10 mg/kg of TGN or vehicle solution was orally administered to 5XFAD mice for 14 days. The time schedule of the experiment is shown in Fig. 3A. Body weights were recorded during drug administration, and no significant changes were observed among the groups (Fig. 3E). In the open field test, no significant locomotor differences were detected in the distance moved between the groups (Fig. 3D). In the object recognition memory test (Fig. 3B), all mice showed equivalent exploratory behaviour toward each of the objects (preference indices were approximately 50%) in the training session. In the test session performed after a 1-h interval, wild-type mice showed a significant increase in recognition of the novel object. In contrast, vehicle solution-treated 5XFAD mice could not recognise the object. However, treatment with TGN significantly increased their object recognition memory. An object location memory test was performed to test spatial memory (Fig. 3C). The results indicated that TGN-treated 5XFAD mice significantly increased their exploratory behaviour toward the object in a novel location.

### A significant increase in neurofilament light chain expression following oral administration of TGN

**Figure 4:**
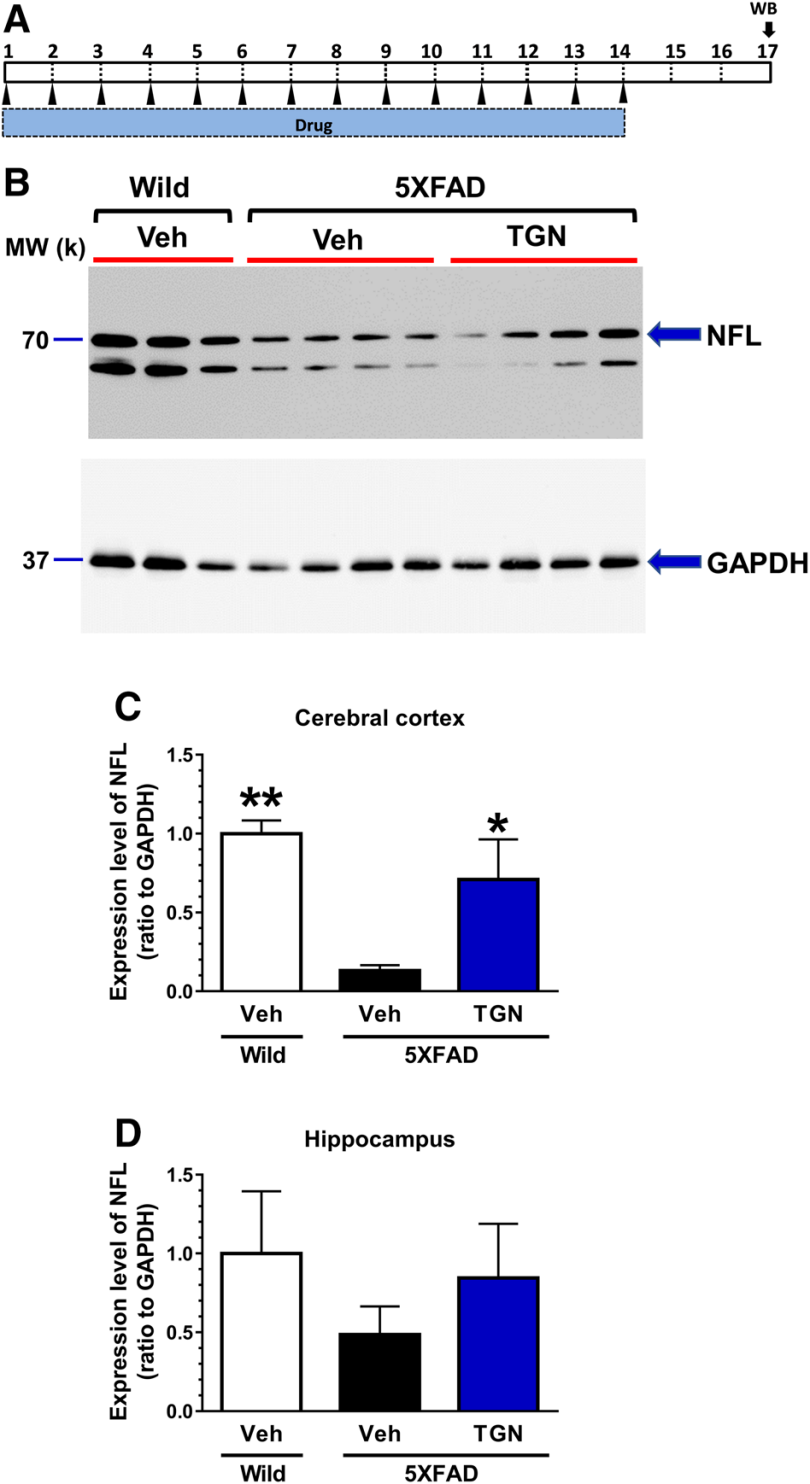
Effects of trigonelline (TGN) on neurofilament light (NFL) levels in the brain. (**A**) Time course of the experiment. (**B**) Representative NFL expression in the cerebral cortex by western blotting. Bands at 70 k were quantified as NFL according to the manufacturer’s datasheet. As a loading control, glyceraldehyde-3-phosphate dehydrogenase (GAPDH) expression was also measured. (**C**,**D**) Quantitative values of the expression levels of NFL (ratio to GAPDH) are shown for the cerebral cortex (**B**) and hippocampus (**D**). The statistical analysis was performed using a one-way analysis of variance (ANOVA) and post hoc Dunnett’s test, *P < 0.05, **P < 0.01 versus Veh-treated 5XFAD mice, n = 6–7 mice.

It is well known that level of NFL is decreased in the brain associated with axonal disruption, and NF-L level in plasma is conversely increased. In the present study, 3 days following the 14-day oral administration of TGN (10 mg/kg) or vehicle solution (water) to 5XFAD model mice, the cerebral cortex and hippocampus lysates were prepared and subjected to western blotting analysis. The time schedule of the experiment is shown in Fig. 4A. NFL levels were low in the vehicle solution-treated 5XFAD mice compared to the wild-type mice. The TGN-treated group showed a significant increase in NFL levels in the cerebral cortex (Fig. 4B,C) and a trend of an increase in that in the hippocampus (Fig. 4D).

Presynaptic marker, synaptophysin was measured in the cerebral cortex (Supplementary Fig. 2). Compared wild-type mice, vehicle-treated 5XFAD mice showed a trend of decrease in synaptophysin. Although synaptophysin in TGN-treated 5XFAD tended to be increased, no significant different among 3 groups. Expression level of Aβ oligomers in the cerebral cortex was evaluated (Supplementary Fig. 3). Wild-type mice expressed neither Aβ oligomers nor human APP. Drastically increased level of Aβ oligomers in 5XFAD mice was not changed by TGN treatment.

### Creatine kinase B-type is the binding target of TGN in neurones

**Figure 5:**
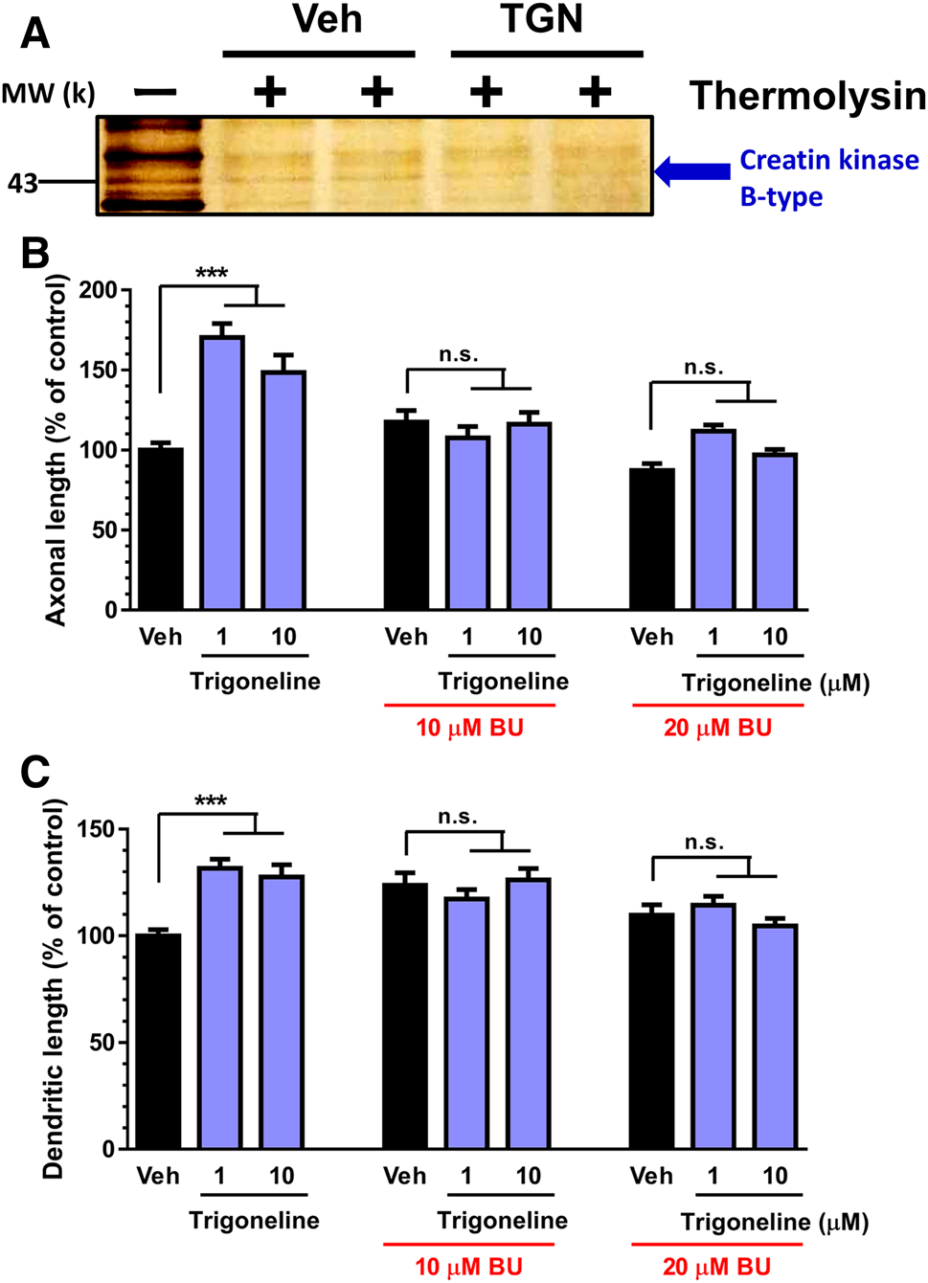
Creatine kinase B-type was identified as a binding target of trigonelline (TGN). (**A**) A drug affinity responsive target stability (DARTS) analysis of the mouse cortical neurones was performed using lysates of the cultured cortical neurones. After treatment with vehicle solution (water) or 100 µM TGN for 30 min at room temperature, thermolysin treatment was added. At 43 K, the band level in the TGN-treated lysates was lower than that in the vehicle solution-treated group. The protein band was cut and analysed by nano liquid chromatography-mass spectrometry. The images in the figure were cropped, and the full-length gel is presented in Supplementary Fig. 2. (**B**,**C**) After the cortical neurons were cultured for 3 days, BU99006, as an inhibitor for creatine kinase B-type (concentrations: 10, 20 µM) was treated with TGN (doses: 1 and 10 µM) for 4 days. Then, the axons and dendrites were immunostained. Phosphorylated neurofilament-H (pNF-H) and microtubule-associated protein 2 (MAP2) were stained as axonal and dendritic markers, respectively. The statistical analysis was performed using a one-way analysis of variance (ANOVA) and post hoc Bonferroni’s multiple comparison test, ***P < 0.001 versus Veh, n = 12–15 areas.

We explored the mechanism of action of TGN, which mediates axonal and dendrite outgrowth. For a comprehensive analysis, a drug affinity responsive target stability (DARTS) analysis was performed as described previously^11,12^. The band intensity of a protein around 43 kDa was thinner in the TGN-treated lysate than in the vehicle-treated lysate (Fig. 5A, Supplementary Fig. 4). The protein band was excised, digested with trypsin, and identified by nano LC–MS/MS with searching in the MASCOT database. The band was identified as creatine kinase B-type (protein sequence coverage: 45%, score: 616).

The involvement of creatine kinase B-type in TGN-induced phenomena was investigated. TGN (1 and 10 μM) extended the lengths of axons and dendrites in normal (no Aβ treatment) cortical neurones (Fig. 5B,C). When TGN was applied to neurones with BU99006, an inhibitor of creatine kinase B-type, the TGN treatment did not increase the lengths of axons and dendrites (Fig. 5B,C). This suggests that the TGN-induced neurite outgrowth effect is mediated by creatine kinase B-type neurones.

## Discussion

### Anti-AD effects of TGN

The reconstruction of neuronic networks after brain damage is one of the target areas considered in the development of new drugs for neurodegenerative diseases. In the present study, we confirmed that TGN could ameliorate axonal and dendritic atrophy in Aβ-treated cortical neurones (Fig. 1), which is in agreement with our previous study^15^. Although other groups focussed on the neuroprotective effect of TGN, at least 5XFAD mice showed a very slight neuronic loss in a limited area, and synaptic loss mainly proceeds neuronic death^19^. In this study, the level of the main axonal component, NFL, in the cerebral cortex was lower in 5XFAD mice than in wild-type mice. TGN administration for 14 days recovered the NFL levels (Fig. 4B,C), suggesting that axonal disruption in the brain of 5XFAD mice might be ameliorated by TGN treatment. Memory dysfunction was also improved by TGN administration in 5XFAD mice (Fig. 3B,C). Neurofilaments are the most important cytoskeletal proteins in myelinated axons and consist of four subunits: neurofilament light (NFL; MW 67–69 k), neurofilament medium (NFM; MW 145–160 k), and neurofilament heavy (NFH; MW 200 k) chains together with α-internexin. NFL is a component of the most abundant intermediate filament primarily expressed in large-calibre myelinated axons^12^. In animal studies, NF-L levels have been used as a marker of axonal damage for many years^20^. After axonal disruption, NFL diffuses easily from the parenchyma into the cerebrospinal fluid (CSF). Elevated levels of NFL in either the CSF or serum are often used as potential biomarkers of axonal injury, axonal loss, and neuronic death in the case of AD^21^. Animal studies have shown that NFL expression levels in the brain are lower in AD model mice than in wild-type mice^22^.

Aβ level in the cerebral cortex wasn’t changed by TGN administration, suggesting that memory recovery by TGN is not mediated by Aβ lowing (Supplementary Fig. 2). Presynapse marker, synaptophysin level was not significantly changed by TGN although a trend of increase was shown (Supplementary Fig. 3). Decrease in synaptophysin level in 5XFAD mice was not clearly detected at least by western blotting as other reports showing^23,24^. More fine detection of synaptophysin at limited presynaptic areas might be needed in future.

### TGN signalling in neurons

TGN showed these effects in mice by oral administration and orally administered TGN was found to have penetrated the brain (Fig. 2C). Although the TGN transport system in the blood–brain barrier is unknown, the gradual and slow increase in TGN concentration in the brain may indicate sustained and accumulated effects of TGN. Since the direct action of TGN against neurones was clarified by penetrative brain property (Fig. 2C), we explored direct target proteins of TGN in neurones.

To identify the binding target of TGN, we used the DARTS method, which has been used for ligand-target identification. The DARTS method uses alterations in proteolytic sensitivity, which occurs after the binding of the ligand and receptor^11,12^. The greatest advantage of this method is the ability to use the small native molecule without any immobilisation or modification, such as a radioisotope, fluorescent, or photoaffinity labels^25^. Creatine kinase B-type was found to be the target protein of TGN (Fig. 5). The relationship between TGN and creatine kinase B-type has not been previously reported. In addition, the function of creatine kinase B-type related neurite outgrowth has also never been reported. Therefore, the question should be asked—how does creatine kinase B-type regulate neurite extension after binding with TGN? Based on the current knowledge of creatine kinase B-type, this enzyme controls ATP concentrations through the transfer of high-energy phosphate from phosphocreatine to ADP^26,27^. The downregulation of creatine kinase B-type has been reported in numerous neurodegenerative disorders, including AD^28^. An inhibitor of creatine kinase B-type, BU99006, cancelled TGN-induced axonal and dendrite outgrowth. It is unknown whether TGN signalling might alter energy metabolism via creatine kinase B-type, which is affected by neurite formation. However, this might be a potential explanation.

The bioavailability and transferability of the brain are important factors in the development of central nervous system drugs. Although safety is also crucial as the premise for drug development, the safety of TGN has already been described in other experiments. For example, the oral and subcutaneous LD50 doses of TGN is 5000 mg/kg in rats, and it causes no acute toxicity and mutagenesis in mice^29^. The properties of TGN, such as that it is orally available, safe and can penetrate the brain, make it an attractive candidate for future central nervous system medicine.

In conclusion, we found for the first time that TGN penetrates the brain and may activate creatine kinase B-type, leading to axonal formation. This study shows the potential of TGN as a new drug candidate, and a new target molecule, creatine kinase B-type, in memory recovery signalling.

## Supplementary information

Supplementary Figures.

## Data Availability

All data needed to evaluate the conclusions in the paper are present in the paper or the Supplementary Materials.
