# Supplementary material for: Trigonelline recovers memory function in Alzheimer’s disease model mice: evidence of brain penetration and target molecule
Source: Sci Rep. 2020 Oct 2;10:16424. doi: 10.1038/s41598-020-73514-1 (PMC7532147; doi:10.1038/s41598-020-73514-1)
Supplement: Supplementary file 1 — Supplementary Figures. [file 41598_2020_73514_MOESM1_ESM.pdf]

Supplementary Information

Trigonelline recovers memory function in Alzheimer's disease model mice: Evidence of brain penetration and target molecule

Mai M. Farid[1,2], Ximeng Yang[1], Tomoharu Kuboyama[1], Chihiro Tohda[1]*

[1]Section of Neuromedical Science, Division of Bioscience, Institute of Natural Medicine, University of Toyama, Toyama, Japan
[2]Pharmaceutical and Drug Industry Division, Department of Phytochemistry and Plant Systematics, National Research Centre, Giza, Egypt

*Correspondence: Chihiro Tohda, Ph.D.
Section of Neuromedical Science, Division of Bioscience, Institute of Natural Medicine, University of Toyama, 2630 Sugitani, Toyama 930-0194, Japan
E-mail: chihiro@inm.u-toyama.ac.jp
Phone: +81-76-434-7646

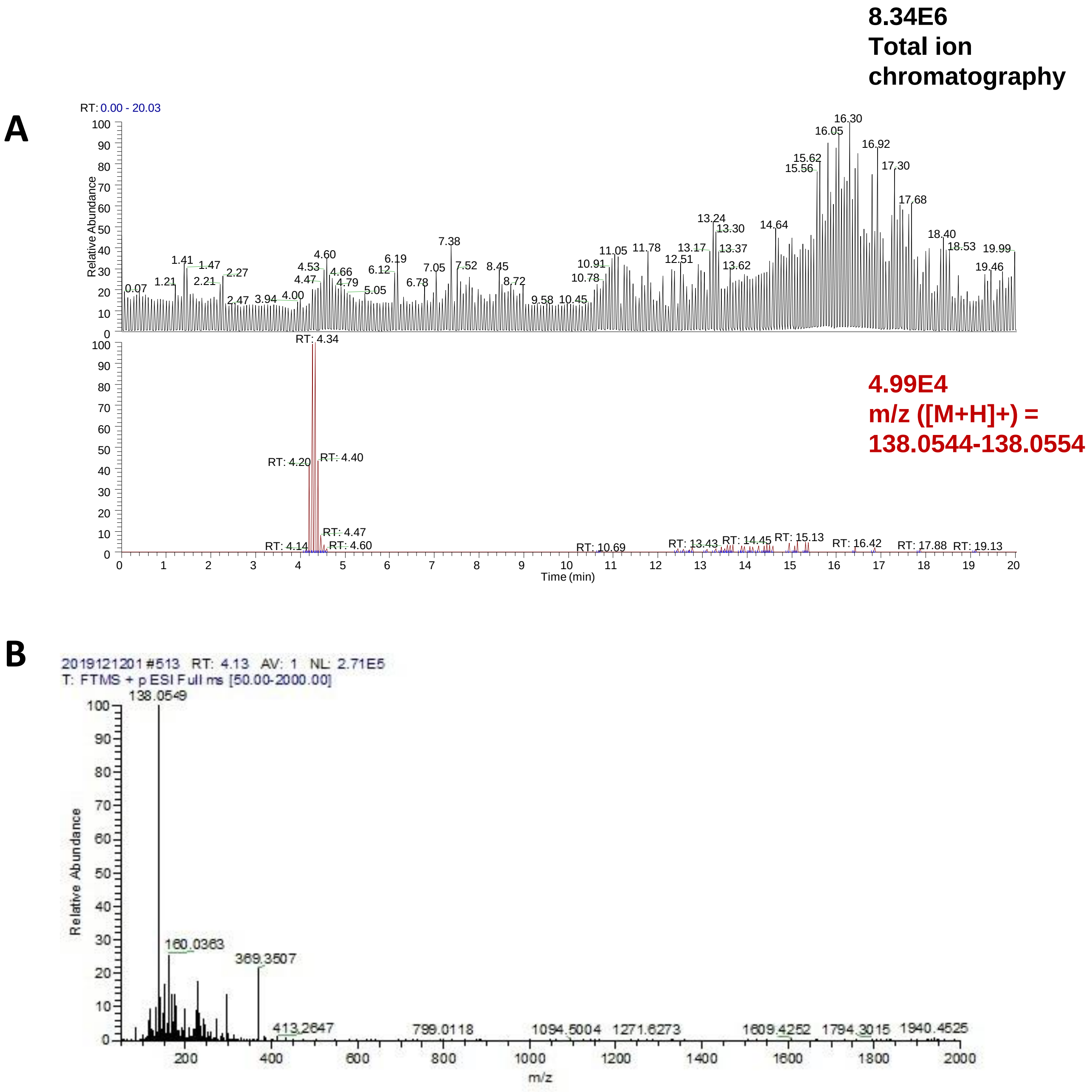

Supplementary Figure 1

Total ion chromatogram (A) and extracted ion current chromatogram (B) of standard TGN (m/z 138.0549) at 10 µg/ml. Fragmentation pattern of MS/MS data was shown in (C).

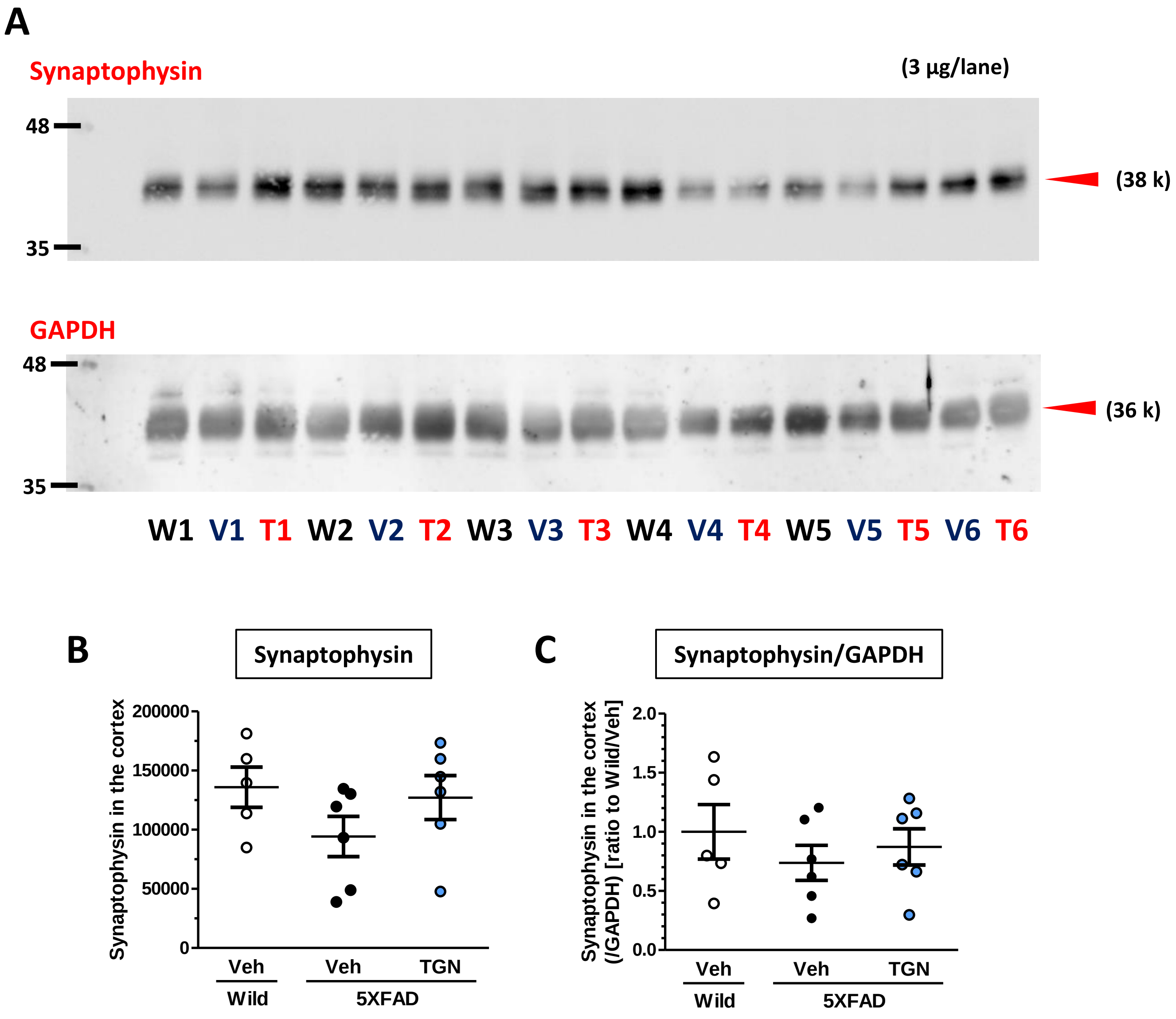

Supplementary Figure 2

Effects of trigonelline (TGN) on synaptophysin levels in the cerebral cortex.
(A) Representative synaptophysin and a loading control, glyceraldehyde-3-phosphate dehydrogenase (GAPDH) expression expression in the cerebral cortex by western blotting. Quantitative values of the expression levels of synaptophysin (B) and ratio to GAPDH (C) are shown. The statistical analysis was performed using a one-way analysis of variance (ANOVA) and post hoc Dunnett's test, $^{*}P < 0.05$, $^{**}P < 0.01$ vs Veh-treated 5XFAD mice, n = 5 – 6 mice.

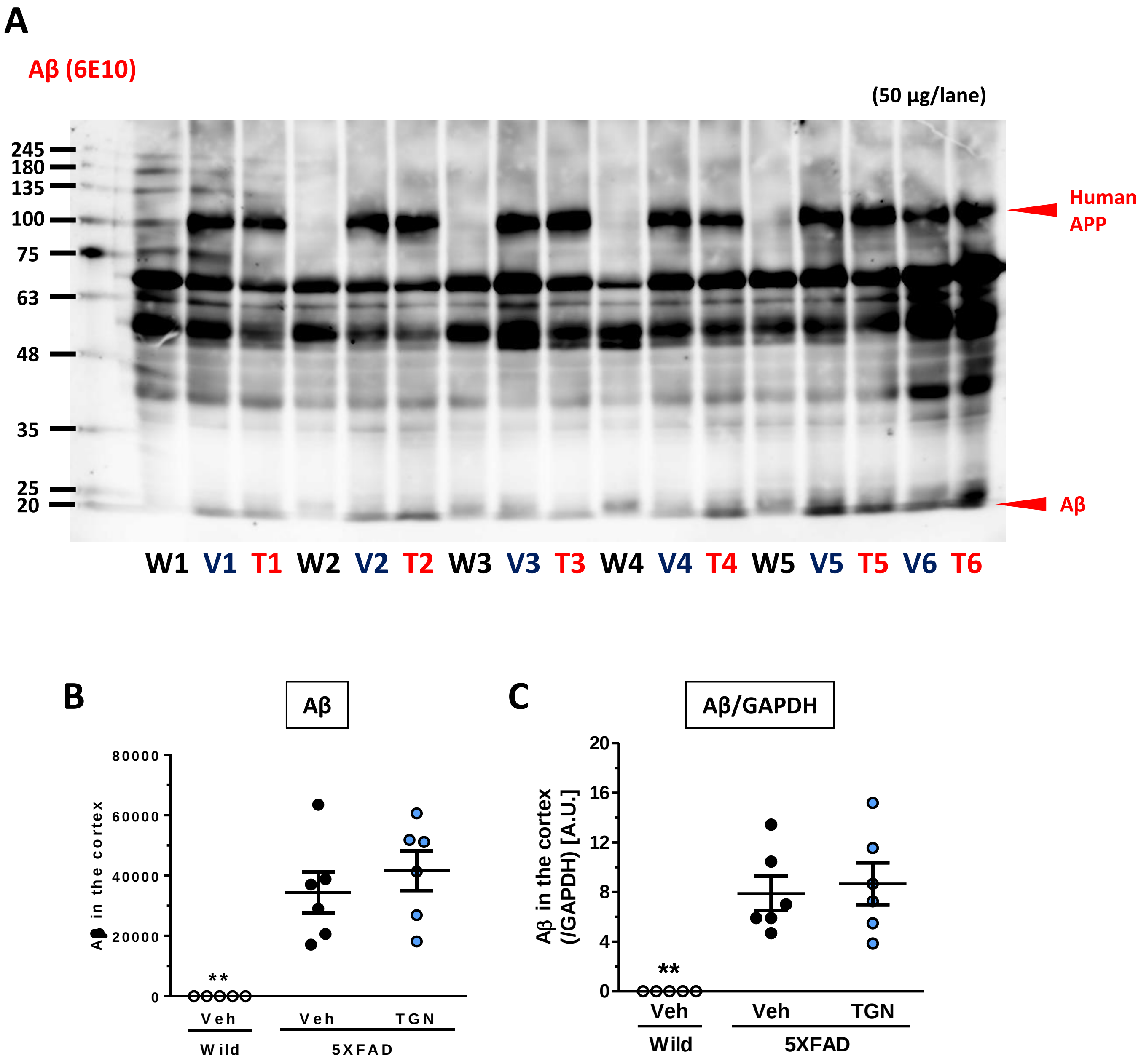

Supplementary Figure 3

Effects of trigonelline (TGN) on Aβ oligomer levels in the cerebral cortex.
(A) Representative Aβ oligomer expression in the cerebral cortex by western blotting. Quantitative values of the expression levels of Aβ oligomer (B) and ratio to GAPDH (C) are shown. Overexpressed human APP was detected only in 5XFAD mice. The statistical analysis was performed using a one-way analysis of variance (ANOVA) and post hoc Dunnett's test, **$P < 0.01$ vs Veh-treated 5XFAD mice, n = 5 – 6 mice.

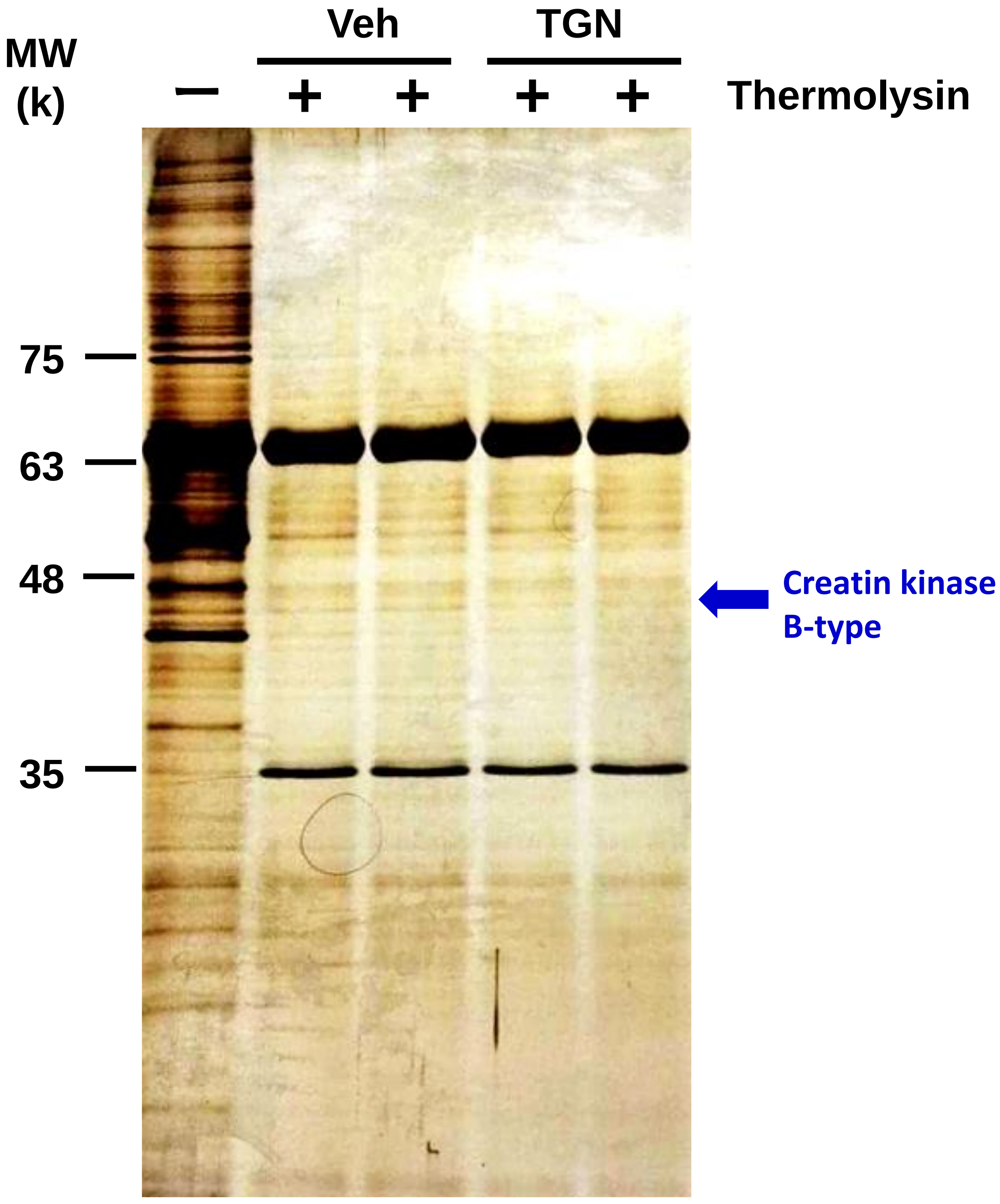

Supplementary Figure 4

DARTS analysis of mouse cortical neurons was performed using lysate of cultured cortical neurons. After treatment with vehicle solution (water) or 100 µM TGN for 30 min at room temperature, thermolysin treatment was added. Below 48 k, the band level in TGN-treated lysates was thinner compared with the vehicle solution-treated group.
